# The Influence of Neighborhood on Infant Parasympathetic Nervous System Development

**DOI:** 10.1002/dev.70074

**Published:** 2025-09-02

**Authors:** Marisa N. Lytle, Anna M. Zhou, Elizabeth Youatt, Centia Thomas, Dawn Witherspoon, Vanessa LoBue, Kristin A. Buss, Koraly Pérez‐Edgar

**Affiliations:** ^1^ Department of Psychology The Pennsylvania State University University Park Pennsylvania USA; ^2^ Department of Psychiatry University of Colorado Anschutz Medical Campus Aurora Colorado USA; ^3^ Department of Psychology University of Denver Denver Colorado USA; ^4^ Department of Psychology Eastern Michigan University Ypsilanti Michigan USA; ^5^ Department of Psychology Rutgers University‐Newark Newark New Jersey USA

**Keywords:** infancy, maternal depression, neighborhood, respiratory sinus arrhythmia, social support

## Abstract

The adaptive calibration model (ACM) asserts that the stress response system, including the parasympathetic nervous system (PNS), conditionally adapts to one's environment. In infancy, the proximal context of parental influences (e.g., maternal depression/anxiety) affects the development of the infant stress response system. In contrast, the relation between broader contexts, such as neighborhood environment, and infant PNS development is less well understood, despite the impact neighborhoods may have on maternal mental health and youth outcomes. The present study bridges prior research examining relations between neighborhood and maternal depression, and maternal depression and infant PNS development. Latent growth curve analysis with mother–infant dyads (*N* = 320), assessed when infants were 4, 8, 12, 18, and 24 months old, indicated that maternal depressive symptoms showed global decreases across infancy and that infant resting RSA showed global increases, as well as variability across the sample in these trajectories. Moderated mediation modeling showed significant direct effects of neighborhood structural disadvantage on initial levels of infant resting RSA. However, there were no significant indirect effects of neighborhood through maternal depressive symptoms on RSA levels or trajectories. These findings suggest that the broader environment impacts infant stress response system development, but this may occur through other mechanisms beyond proximal maternal depressive symptoms.

## 1 Introduction

Exposure to environmental adversity in periods marked by rapid physiological development may “get under the skin” to influence long‐term physiological functioning (Boyce and Ellis 2005). One way in which contextual factors may influence physiological development is by calibrating the individual's stress response system, including both basal levels and reactivity to stress, as proposed by the adaptive calibration model (ACM; Del Giudice et al. 2011). The ACM asserts that conditional adaptation to stressors in one's environment results in differential tuning of the stress response system, making it more or less susceptible to environmental influence. This fine‐tuning of the stress response system has the potential to be evolutionarily adaptive for operating within the contexts that individuals experience in their everyday lives (Boyce and Ellis 2005).

The current study examined if and how neighborhood context may calibrate the parasympathetic nervous system (PNS) branch of the stress response system in infancy. Neighborhood stress is not often examined as a direct impact on the daily lives of infants despite growing understanding that development must be situated within context, including both the home and the broader environment (Bolger et al. 1988). Much of the prior literature has focused on proximal influences on PNS activity, often in the form of maternal traits. For example, increased maternal depression has been associated with lower levels of infant resting PNS activity (Jacob et al. 2009; K. C. Johnson et al. 2014). However, we know that the larger environment, such as the neighborhood, shapes the contexts that infants must adapt to (Bronfenbrenner and Ceci 1994). While there may be direct effects, maternal traits and behaviors with respect to their infant may also reflect the caregiver's experience of the daily environment (Kohen et al. 2008; May et al. 2018). Therefore, the current study also aimed to investigate the mediating role of maternal depressive symptoms in the relation between neighborhood and infant PNS development, given that maternal traits have a robust impact on infant trajectories.

### 1.1 PNS Developmental Trajectory

One of the primary components of the stress response system is the autonomic nervous system (ANS), within which the sympathetic nervous system (SNS) is often thought to be responsible for responding to stressors (flight or fight) and the PNS is responsible for recovery (rest and digest) (McCorry 2007). However, the polyvagal theory proposes that the PNS may play an important part in responding to challenge or low‐level stressors (Porges and Furman 2011). Specifically, both the SNS and PNS can move in two directions independently, presenting increasing and decreasing activity. When task or stressor demands are perceived as relatively minor, the body may be able to cope with these demands by decreasing PNS influence alone, rather than also increasing SNS activity (Porges 2007).

Vagal tone is a measure of PNS influence over the heart and is primarily indexed by respiratory sinus arrhythmia (RSA), a measure of high‐frequency heart rate variability that oscillates with spontaneous breathing (Porges and Furman 2011). High‐frequency heart rate variability has been found to be almost entirely controlled by the vagus nerve, which provides parasympathetic control over the heart from the brain (Berntson et al. 1997). High levels of resting RSA have been interpreted as reflecting the ability for an individual's nervous system to quickly and positively respond to stressors in their environment and regulate behavior and emotion elicited by the stressor, as higher levels allow more room to withdraw PNS control from baseline to task (Calkins 1997). Measures of resting PNS activity are relatively stable after 1 year of age and reflect a trait‐level ability to regulate one's response to stress (Alkon et al. 2011; Dollar et al. 2020; Wagner et al. 2021).

Recent work has sought to map normative resting RSA trajectories to determine sensitive periods in development (Dollar et al. 2020; Wagner et al. 2021). Although studies have varied in the age range investigated, vagal tone shows rapid increases during the first year of life (Bar‐Haim et al. 2000; Porges and Furman 2011). Further, vagal tone shows relative instability in the first 6 months such that infant RSA measured at 1 month has shown no associations with RSA measured at 3 or 6 months (Porter et al. 1995). Averaging across individuals, resting RSA continues to increase in the first 3 years of life. However, there is also variability in the degree of increase, which leaves room for exploring potential predictors of differences in rates of change over time (Wagner et al. 2021). Following this initial increasing period, resting RSA plateaus between 4 and 10 years of age and shows greater levels of stability (Alkon et al. 2011; Bar‐Haim et al. 2000; Bornstein and Suess 2000; Dollar et al. 2020).

Developmental trajectories of resting RSA show variability in increases across the first few years of life, indicating the potential for external influence on trajectories in this time of rapid change. Context may play an important role in shaping the development of resting RSA as a marker of the capacity to respond to stress. Indeed, according to the ACM (Del Giudice et al. 2011), environments marked by low levels of stress may relate to the development of higher basal vagal tone, promoting high flexibility in response to environmental demands. Across individuals, as environmental stress increases, levels of basal vagal tone may decrease, potentiating the stress response system to allow for faster or greater sympathetic dominance in response to stress (Del Giudice et al. 2011). Resting RSA, potentially calibrated by environmental and genetic input early in life, then serves as a marker of susceptibility to context in the future. Empirical work in relatively high‐risk samples has found that infants lower in resting RSA may be more susceptible to differences in maternal depression (Somers et al. 2019) and neighborhood characteristics (Zhang et al. 2018) in relation to later child behavioral outcomes. Given this work indicating the importance of resting RSA as a potential marker of susceptibility to context, it is important to consider how differences in context may calibrate vagal tone to be best suited to the potential stressors an infant may encounter.

### 1.2 Theories of Neighborhood Influence on Child Outcomes

According to Bronfenbrenner's ecological systems theory, children develop within a set of nested contexts that influence the child directly, as well as through transactional influences between contexts (Bronfenbrenner and Ceci 1994). For example, the nuclear family is conceptualized as situated in the microsystem, the innermost context, and has direct daily interactions with the child. In contrast, in infancy, neighbors are situated in the exosystem as they primarily interact with caregivers and influence the child through the mesosystem, that is, transactional influences between the neighbor, caregiver, and child.

Within research on RSA development, maternal and familial influences within the microsystem have thus far been the primary context of interest. However, a wealth of literature has called attention to the importance of studying neighborhood context given its influence on child outcomes across domains of development (Anderson et al. 2014; Leventhal and Brooks‐Gunn 2000; D. P. Witherspoon et al. 2023), including in early childhood (Ma and Grogan‐Kaylor 2017; Pei et al. 2022).

The prevailing theory utilized to explore influences of negative neighborhood characteristics on youth outcomes is social disorganization theory (Sampson et al. 2002; Shaw and McKay 1942). This deficit‐focused model examines negative neighborhood structural characteristics, such as poverty, residential instability, and increased percentage of single‐parent households, often grouped under the construct of neighborhood disadvantage (Sampson et al. 1997). The argument is that these structural deficits negatively influence the ability of neighbors to form collective trust and cohesion (Shaw and McKay 1942). Lack of social cohesion, as well as lack of established neighborhood social norms, is theorized to result in youth not having adequate role models and supervision, which in turn results in negative outcomes, such as increased rates of crime and mental illness, as well as higher rates of maladaptive outcomes in youth (Kim 2010; Kingston et al. 2009; Leech 2016; Lei and Beach 2020; Leventhal and Brooks‐Gunn 2000; Leventhal and Dupéré 2019; McBride Murry et al. 2011)

Despite empirical findings motivated by social disorganization theory, theorists have called attention to the negative outcome bias and the focus on risk factors and deficits in disadvantaged neighborhoods (Aber and Nieto 2000; Wandersman and Nation 1998). In comparison, pluralistic neighborhood theory argues that neighborhoods labeled as structurally disadvantaged also have protective factors that are associated with positive youth outcomes (Aber and Nieto 2000). These strengths include social resources such as informal social control and social cohesion, which are often grouped together under the construct of collective efficacy (Sampson et al. 1997). Informal social control refers to the likelihood that adults in the neighborhood will intervene and monitor youth behavior, and social cohesion refers to the strength of social bonds and trust between neighbors (Sampson et al. 1997).

Pluralistic neighborhood theory suggests, and recent empirical work following this approach find, that within a given neighborhood, perceptions are heterogeneous and that these perceptions have implications for outcomes (Browning et al. 2014; Butler et al. 2012; D. Witherspoon and Ennett 2011). However, it is important to note that these findings are not always consistent across samples with varying racial/ethnic composition (see White et al. [2021] for review). Using pluralistic neighborhood theory as a guiding framework, it is important to not only seek to investigate how neighborhood structural characteristics relate to outcomes but also explore how protective factors, such as collective efficacy, may operate within these neighborhoods to influence developmental outcomes.

### 1.3 Neighborhood Influence on Maternal Psychopathology

According to Bronfenbrenner's ecological systems theory, exosystem contexts such as the neighborhood will influence youth through the microsystem contexts, such as family (Bronfenbrenner and Ceci 1994), particularly for infants and children. This may hold especially true for infants who cannot yet walk and explore their neighborhood and the environment outside of the home (Leventhal and Brooks‐Gunn 2000). It is thus important to consider that the family may play a mediating role in conferring neighborhood risk and protective factors to infant outcomes.

Importantly, theoretical and empirical evidence supports that neighborhood stressors influence maternal mental health outcomes (May et al. 2018; McCloskey and Maguire‐Jack 2021), which have been shown to impact infant PNS development (de Vente et al. 2020; Zhou et al. 2023). Wandersman and Nation (1998) theorize that neighborhood contextual characteristics (e.g., neighborhood SES, racial/ethnic composition, residential patterns) in combination with low social support may be associated with adult mental health. In line with this model, increased neighborhood structural disadvantage (Hill and Herman‐Stahl 2002; Kim 2010; Kohen et al. 2008; Manuel et al. 2012; May et al. 2018; Ross 2000), as well as maternal perceived neighborhood disorder (McCloskey and Maguire‐Jack 2021), is associated with maternal depression. In addition, in support of pluralistic neighborhood theory, neighborhood support (Kim and Ross 2009), as well as support networks and partner support (Manuel et al. 2012; McCloskey and Maguire‐Jack 2021), moderates the relation between neighborhood and maternal depression.

Not only are neighborhood social supports related to maternal psychopathology, but they are also associated with positive parenting quality. Specifically, Rhoad‐Drogalis et al. (2020) found that greater levels of social cohesion and control were associated with higher levels of parental involvement in the first year of infancy. These studies investigating the influence of neighborhood on maternal symptomatology and parenting behaviors are important due to the insight they may provide into the transactional influences (i.e., Bronfenbrenner's mesosystem) between the neighborhood and the mother.

### 1.4 Maternal Psychopathology and RSA Development

While we lack a deep literature on the effect of neighborhood context on infant PNS development, there is prior work indicating that proximal factors, such as maternal sensitivity and maternal depression, influence the trajectory of infant RSA (Propper and Holochwost 2013; Tacana et al. 2024). Specifically, in both low‐income and middle‐to‐high‐income samples, maternal depression has been associated with lower levels of infant resting RSA (Jacob et al. 2009; K. C. Johnson et al. 2014), and maternal sensitivity in infancy has been associated with higher levels of resting RSA at 5 years of age (M. Johnson et al. 2017) and smaller increases in resting RSA from 7 to 18 months of age (Tacana et al. 2024). These studies investigate static levels of maternal depressive symptoms and sensitivity in infancy. However, mothers show variation not only in their levels of depressive symptoms but also in how these symptoms change over time (Wu et al. 2011). Children whose mothers showed slower rates of decline in depressive symptoms across infancy were found to have poorer social skills (Wu et al. 2011) and more behavioral difficulties in early childhood (van der Waerden et al. 2015). Together, the emerging data reinforce the importance of studying how different rates of change in maternal depressive symptoms across infancy relate to infant regulatory development (van der Waerden et al. 2015).

Caregivers play a primary role in shaping infant experiences and development. However, infants also play an active role in eliciting behaviors from their caregivers (Bell and Harper 1977; Cohn and Tronick 1987; Curci et al. 2023; Somers et al. 2021; Vallotton 2009). Maternal behavior influences child outcomes but, at the same time, infant behavior and/or physiology may also affect change in maternal behavior. For example, Curci et al. (2023) found that infant behavior problems at 12 months of age significantly predicted increased maternal depressive symptoms measured at 18 months, controlling for maternal depressive symptoms at 12 months. These recent findings point toward the importance of considering bidirectional relations between infant characteristics and changes in maternal symptomatology across infancy.

Despite the theoretical understanding that neighborhood may impact youth by way of neighborhood–parent and parent–child interactions, no empirical studies investigate this pathway with regard to infant RSA. Prior work has investigated the role of maternal depression and parenting in shaping the relation of neighborhood disadvantage and toddler behavioral outcomes and found support for both the mediating and moderating roles of maternal depression (Fuller et al. 2018; Kohen et al. 2008). In particular, Kohen et al. (2008) found an indirect positive relation between neighborhood structural disadvantage and a broad index of toddler behavior problems through neighborhood cohesion, maternal depression, and punitive parenting, such that increased neighborhood disadvantage was related to increased behavior problems. As resting RSA is conceptualized as a physiological measure of regulation that is associated with behavioral outcomes, these findings provide support for exploring the influence of neighborhood on infant RSA outcomes.

### 1.5 The Present Study

The present study examines the potential influence of neighborhood on infant RSA trajectories. Additionally, this study investigates how maternal depression may mediate associations between neighborhood and infant PNS development. The present study focuses on developmental trajectories across infancy due to the rapid increases in RSA levels across this period (Dollar et al. 2020; Wagner et al. 2021), building on prior work showing that steady high or increasing maternal depressive symptoms may relate to increases in RSA reactivity (Ashman et al. 2008).

Based on prior literature that has investigated the indirect pathways of neighborhood characteristics to maternal depression and maternal depression to infant emotional regulation (Kohen et al. 2008), we hypothesize that neighborhood structural disadvantage would have an indirect effect on infant RSA. This effect would be mediated through initial levels of maternal depressive symptoms, such that higher levels of neighborhood disadvantage would be associated with increased initial levels of maternal depressive symptoms. These increased initial levels of maternal depressive symptoms would then be related to decreased initial levels of resting RSA, as well as smaller increases in resting RSA over time. Furthermore, we hypothesized that higher levels of neighborhood disadvantage and lower levels of neighborhood support would also result in smaller decreases in maternal depressive symptoms across infancy, which would also relate to smaller increases in resting RSA as infants adapt to their context. In addition, in line with pluralistic neighborhood theory (Aber and Nieto 2000), we hypothesized that parental perceived collective efficacy would moderate the relation between neighborhood perceived disadvantage and maternal depressive symptoms, as neighborhood social support and informal control may decrease initial levels of maternal depressive symptoms as well as change in maternal depressive symptoms across infancy.

## 2 Materials and Methods

### 2.1 Participants

Data for the present study were collected as part of the larger Longitudinal Attention and Temperament Study (Pérez‐Edgar et al. 2021). In this study, data were collected from infants (*N* = 357) longitudinally at 4, 8, 12, 18, and 24 months of age, using a multimethod approach that included caregiver questionnaires assessing infant temperament, their own psychological state and traits, and the sociodemographic features of their environment, as well as experimental measures of infant attention, reactivity, and physiological response. Initial participant recruitment took place from 2016 to 2019, with final in‐person data collection concluding in 2020, due to COVID mitigation. The Institutional Review Boards at The Pennsylvania State University and Rutgers University approved all procedures, and parents provided written consent and were compensated for their participation.

Participants were recruited from areas surrounding three sites: State College, PA (*N* = 167); Harrisburg, PA (*N* = 81); and Newark, NJ (*N* = 109). Caregivers identified 16% of the infants as African American/Black, 3% as Asian, 22% as Latinx, 50% as White, and 8% as biracial or mixed race. Across the sample, 14% of families reported a household income of $15,000 or less, 6% reported $16,000–$20,000, 6% reported $21,000–$30,000, 5% reported $31,000–$40,000, 6% reported $41,000–$50,000, 8% reported $51,000–$60,000, and 39% reported an income above $60,000. The remaining 17% of caregivers declined to provide income information.

The proposed study focuses on a subsample of infants (*N* = 320; 164 female) who completed at least one measure from the current study and for whom the mother was the questionnaire respondent.

### 2.2 Procedures

Demographic questionnaires as well as community survey questionnaires used in the present study were administered during the 4‐month timepoint, either online prior to the lab visit or while infants were completing attention tasks. Similarly, mothers completed the questionnaire to assess maternal depressive symptoms at each visit either prior to coming to lab or while infants were completing attention tasks. Infant resting RSA was collected during the second lab visit of the 8‐, 12‐, 18‐, and 24‐month timepoints.

### 2.3 Measures

#### 2.3.1 Resting RSA (8, 12, 18, and 24 Months)

Electrocardiograph (ECG) signal from the infant was continuously recorded during a resting state at each of the 8‐, 12‐, 18‐, and 24‐month timepoints. Gelled sensors (stickers) were placed by the experimenter on the child's right collarbone and lower left and right ribs prior to baseline. The resting period was 4 min in duration, and the infant was positioned on a parent's lap and given nonstimulating toys to keep them occupied. Parents were instructed to avoid social contact and keep as neutral as possible. ECG was sampled at a rate of 500 Hz using Mindware MW1000A devices and the BioLab system (Mindware Technologies, Ltd., Westerville, OH). A device was attached to the back of the highchair or the parent's chair. At the 18‐ and 24‐month visits, the infant wore the device inside a small backpack to allow for locomotor activity for other study procedures.

Data were analyzed offline using the Mindware editing program Mindware HRV, Versions 3.1.4 and 3.1.5, which identified interbeat intervals and detected physiologically improbable intervals using a validated algorithm (Berntson et al. 1990). Trained personnel visually inspected ECG data for R‐peak and artifact identification. RSA was calculated in 30‐s epochs using the 0.240–1.040 Hz power band. Twenty percent of participants were selected to be inspected by a second reviewer. Selection for secondary validation was biased toward files that were identified as having manual edits. Segments that deviated by more than 0.10 were further inspected by both reviewers, and a final value was agreed upon. In the case of messy data due to participant movement, segments that had more than three consecutive beats about which the editor was unsure were discarded. The mean RSA value across all usable epochs for each timepoint was used for analyses. Means are provided in the descriptive analysis results section in Table 1.

**TABLE 1:** Means, standard deviations, and correlations of study variables.

	*N*	*M* (*SD*)	1	2	3	4	5	6	7	8	9	10	11	12	13	14	15	16	17
1. Neigh: Income (in tens of thousands)	251	91.24 (40.40)	1																
2. Neigh: % unemployed	251	4.27 (4.53)	−0.44^*^	1															
3. Neigh: % under poverty	251	11.15 (14.25)	−0.61^*^	**0.50**	1														
4. Neigh: % without HS diploma	251	9.76 (10.10)	−0.52^*^	**0.35**	0.54^*^	1													
5. Neigh: % female headed house	251	14.05 (13.58)	−0.57^*^	0.55^*^	0.75^*^	0.53^*^	1												
6. Perceived neigh disadvantage	232	1.28 (0.51)	−0.35^*^	0.35^*^	0.39^*^	0.46^*^	0.39^*^	1											
7. Neigh cohesion	240	3.53 (0.71)	0.26^*^	−0.28^*^	−0.27^*^	−0.19^*^	−0.27^*^	**−0.48** ^*^	1										
8. Neigh control	237	3.65 (0.79)	0.30^*^	−0.23^*^	−0.22^*^	−0.16^*^	−0.24^*^	−0.40^*^	0.65^*^	1									
9. Mat. Dep. 4M	234	2.02 (1.33)	−0.09	0.02	0.00	0.10	−0.02	0.08	−0.17^*^	−0.10	1								
10. Mat. Dep. 8M	192	1.70 (1.29)	0.03	0.01	−0.03	−0.01	−0.07	−0.14	−0.01	0.04	0.54^*^	1							
11.Mat. Dep. 12M	158	1.76 (1.47)	0.06	−0.02	−0.15	0.00	−0.06	−0.06	−0.04	0.02	0.52^*^	0.52^*^	1						
12. Mat. Dep. 18M	162	1.79 (1.48)	0.07	−0.07	−0.12	−0.02	−0.16	−0.01	−0.12	−0.10	0.58^*^	0.58^*^	0.61^*^	1					
13. Mat. Dep. 24M	139	1.55 (1.37)	0.00	−0.12	−0.11	−0.02	−0.22^*^	0.06	−0.15	−0.09	0.57^*^	0.57^*^	0.67^*^	0.59^*^	1				
14. Resting RSA 8M	203	3.55 (0.92)	−0.13	0.08	0.10	0.03	0.08	0.09	−0.19^*^	−0.07	−0.03	0.01	0.05	0.11	0.02	1			
15. Resting RSA 12M	152	3.79 (0.90)	−0.07	0.05	0.19^*^	0.07	0.18	0.06	−0.14	−0.03	−0.15	0.08	−0.03	−0.02	0.01	0.50^*^	1		
16. Resting RSA 18M	117	4.01 (1.01)	−0.02	0.04	0.19	0.20	0.20	0.30^*^	−0.17	−0.10	−0.20	−0.13	−0.20	−0.02	−0.10	0.37^*^	0.46^*^	1	
17. Resting RSA 24M	70	4.48 (1.19)	−0.07	0.16	0.13	0.08	0.29^*^	0.10	−0.16	0.03	−0.12	0.14	−0.05	0.03	0.06	0.26^*^	0.61^*^	0.47^*^	1

*p < 0.05.

#### 2.3.2 Neighborhood Structural Disadvantage (4 Months)

Geocoding based on the participant's address collected at the first timepoint was implemented to gather structural components of neighborhood disadvantage. The two‐step process consisted of obtaining the address of the participant and deriving the census block group for that address. Census block group was used in the present analysis to approximate neighborhood characteristics in line with prior literature and due to ease of access to national data (D. Witherspoon and Ennett 2011). The census block group was then entered into socialexplorer.com, which provided sociodemographic variables for the specific geographic location based on the 5‐year estimates of the American community surveys. The 5‐year estimate was selected around the time of participant recruitment, occurring from 2014 to 2018.

Five of the available variables were selected based on use in prior studies (Kohen et al. 2008; Leventhal and Brooks‐Gunn 2000; D. Witherspoon and Ennett 2011) and were derived to measure neighborhood disadvantage. These variables included percentage of residents below the poverty level, percentage of residents aged 16 and older who are unemployed, percentage of residents aged 25 and older without a high school diploma, percentage of female‐headed households, and average neighborhood income. Neighborhood structural disadvantage was then included in the current analysis as a latent factor composed of these five variables. Confirmatory factor analysis on the structural disadvantage latent variable indicated that all variables significantly contributed to the latent variable (see Supporting Information S1).

#### 2.3.3 Neighborhood Perceived Collective Efficacy (4 Months)

The 1994–1995 PHDCN Community Survey (ICPSR 2766) is a survey designed to assess adults’ perceptions of their neighborhood (Sampson et al. 1997) and was collected at the 4‐month timepoint. The survey assesses key neighborhood dimensions, including the dynamic structure of the local community, organizational and political structure, cultural values, social disorder, perceived violence, informal social control, formal social control, and social cohesion. Two components, informal social control and social cohesion, were used to form the combined factor of collective efficacy (Sampson et al. 1997). Informal social control was measured by asking mothers to respond to five statements, such as “If there was a fight in front of your house and someone was being beaten or threatened, how likely is it that your neighbors would break it up?” with a 5‐point scale of *Very unlikely* to *Very likely*. Higher values indicate greater informal social control. Social cohesion was measured by asking mothers to respond to five statements, such as “People in this neighborhood can be trusted,” on a 5‐point scale of *Strongly disagree* to *Strongly agree*. Cronbach's alpha indicated good reliability for the social cohesion (α = 0.87) and social control subscales (α = 0.83). Higher values indicate greater social cohesion.

#### 2.3.4 Neighborhood Perceived Disadvantage (4 Months)

Maternal perceived neighborhood disadvantage was indexed by the social disorder scale of the 1994–1995 PHDCN Community Survey collected at 4 months of infant age (Sampson et al. 1997). This scale consisted of six items that asked mothers to respond to statements such as “How much of a problem is people selling or using drugs?” with a 3‐point scale of *Not a problem* to *A big problem*. Cronbach's alpha indicated excellent reliability (α = 0.91). Higher values on this scale indicate greater maternal perceptions of social disorder.

#### 2.3.5 Maternal Depressive Symptoms (4, 8, 12, 18, and 24 Months)

Mothers self‐reported their depressive symptoms using Beck's Depression Inventory (BDI; Beck et al. 1961) at each study timepoint. The BDI is a 21‐item self‐report questionnaire for evaluating the severity of depression in healthy and psychiatric populations (Beck et al. 1961). Each item consists of a group of related statements, and respondents report how they have been feeling for the past week on a 4‐point scale (0 = *Symptoms absent*, 1 = *Mildly*, 2 = *Moderately*, 3 = *Severe symptoms*), and all items were summed for a total score. Across all timepoints, this measure had good to excellent scale reliability (4 months α = 0.89; 8 months α = 0.86; 12 months α = 0.90; 18 months α = 0.91; 24 months α = 0.92). Higher scores indicate greater symptom severity. Within nonclinical samples, total scores above 20 may indicate depression (Beck et al. 1961). In the current sample, 7% of mothers reported depressive symptoms above this cutoff across all timepoints and 3% at any one timepoint.

### 2.4 Analytic Plan

**FIGURE 1:**
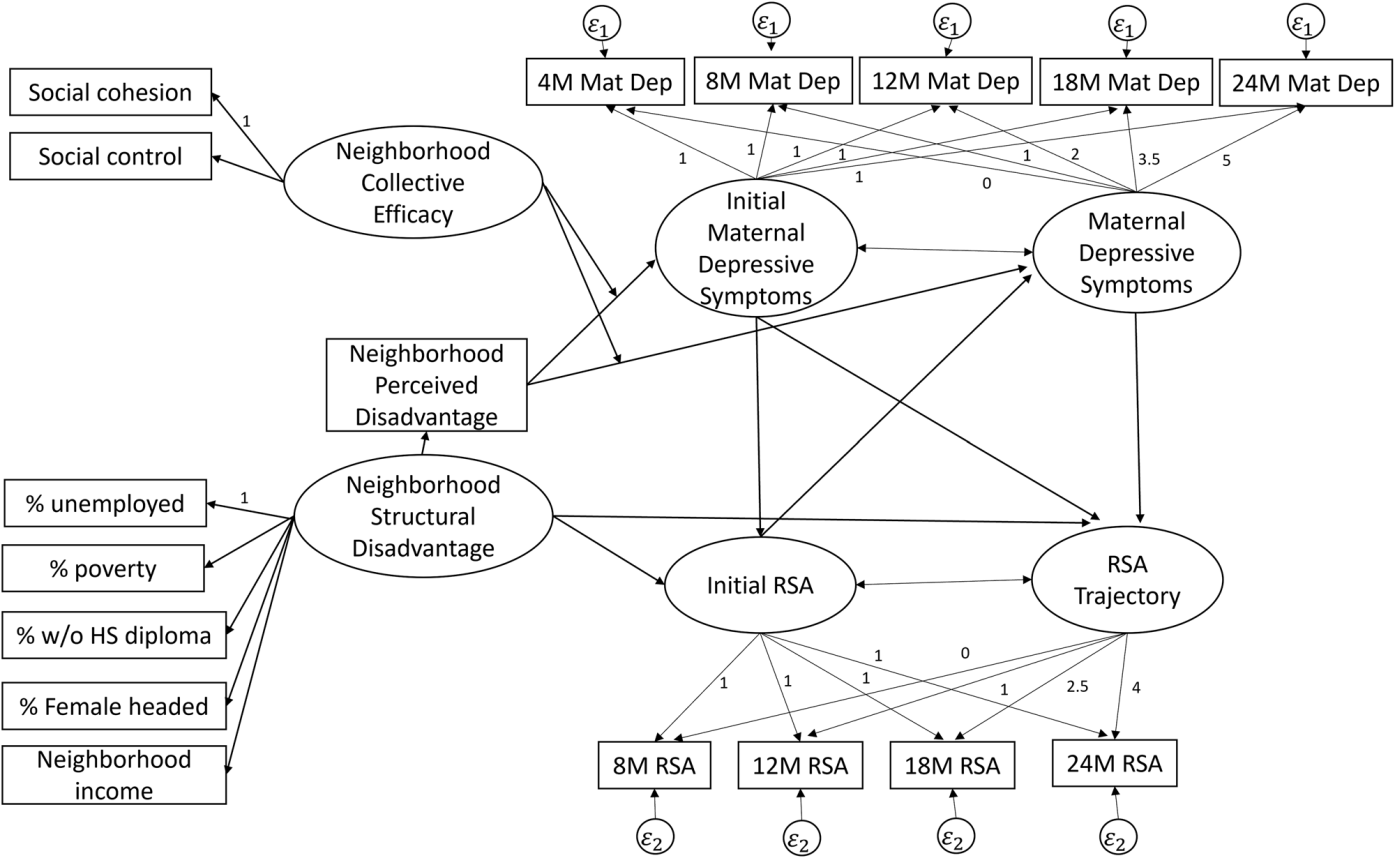
Analytic model.

The proposed analytic model based on the theory‐implied relation between our variables of interest is presented in Figure 1. Structural equation modeling (SEM) was used to test maternal depressive symptoms as a mediator between neighborhood characteristics and infant RSA development. Longitudinal moderated mediation in parallel process latent growth curve (LGC) modeling was selected as the most appropriate modeling technique for the current study, as it allows one to test not only the effect of a mediator but also how change in the mediator affects change in the outcome of interest (Cheong and MacKinnon 2012; Selig and Preacher 2009; Zhu et al. 2021). However, this model relies on key assumptions that must first be tested before testing the final model. Therefore, we began by performing a missing data analysis to ensure bias due to missingness could be minimized with the introduction of covariate or auxiliary variables and full information maximum likelihood (FIML; Collins et al. 2001). We then determined whether there was significant within‐person variability in maternal depressive symptoms and infant resting RSA by calculating intraclass correlation coefficients (ICCs). If ICCs indicated significant variability, individual LGCs were fit to maternal depressive symptoms and infant resting RSA separately to determine if a trajectory model adequately captured the variability in the change across infancy, indicating it could be used as an outcome in the proposed parallel process model.

The proposed analytic model (Figure 1) was then fit to the data, reducing either maternal depressive symptoms or infant RSA to a static composite score if LGCs did not indicate significant variability in change across time between persons. Variables that are identified as potential auxiliary variables were included by covarying the auxiliary variables with all other model variables. Given the importance of isolating neighborhood effects from family and individual level effects, maternal education and child sex were also included as covariates in the model.

Individuals are nested within neighborhoods, which was not accounted for in the analytic model. To handle this potential nesting, ICCs were calculated for all variables as a function of block group to determine the amount of variance within and between neighborhoods. ICCs revealed that a nonzero proportion of variance was attributable to between‐block group differences in maternal depressive symptoms but not infant resting RSA. Maximum likelihood with robust errors was used and compared to results obtained using FIML to account for potentially biased standard errors resulting from the nonindependent structure of the dataset.

All models were estimated using the lavaan package for R (Rosseel 2012). Missing data were accounted for using FIML estimation (Cham et al. 2017). However, missing data corrections such as FIML and multiple imputation are not appropriate in the presence of interactions, as they treat this variable the same as all others and ignore the inherent dependence on the component variables, thus introducing potential bias into parameter estimations (Lüdtke et al. 2020). To handle this potential bias, interaction terms will be removed if nonsignificant, and if significant, models will be examined with and without the term to investigate the potential influence of bias in estimating the nonnormal interaction term under the assumption of multivariate normality. Model fit was assessed using *χ* ^2^, root mean square error of approximation (RMSEA), standardized root mean square residual (SRMR), and Comparative Fit Index (CFI; Hu and Bentler 1999). Cutoff criteria for the fit indices are as follows: *χ* ^2^ *p*‐value > 0.05, RMSEA < 0.08, SRMR < 0.08, and CFI ≥ 0.9. All data and R code for the current study are provided on Databrary (http://doi.org/10.17910/b7.1288).

## 3 Results

### 3.1 Missing Data Analyses

Missingness of demographic and neighborhood variables as well as the outcome measures (maternal depressive symptoms and infant RSA) from the first timepoint is in line with levels commonly seen in studies with children (Ballard et al. 2021). Attrition across timepoints is substantial with 40%–77% missingness in outcome variables at the final two timepoints, largely due to COVID‐related mitigation, which suspended in‐laboratory assessments beginning in March 2020. This level of attrition is more common in randomized control trials (Elobeid et al. 2009) than developmental research (Nicholson et al. 2015). We then tested whether demographic variables, including child sex, maternal education, and site, were appropriate auxiliary variables to help predict missing values in outcome measures across timepoints. Investigation of missing data patterns in outcome measures revealed that maternal education significantly predicted missingness and variation in maternal depressive symptoms and infant RSA across timepoints, a requirement for inclusion of auxiliary variables to account for missing‐at‐random assumptions (Collins et al. 2001).

### 3.2 Descriptive Analyses

Table 1 provides descriptive statistics for all study variables by site. Intraclass correlations revealed that 55% of the variance in maternal depressive symptoms was related to between‐person differences, indicating a significant proportion of individual differences as well as variation across time. In addition, 38% of variability in resting RSA is explained by between‐person differences, also indicating significant individual differences and variation across time.

### 3.3 Model Results

#### 3.3.1 Unconditional Growth Curve Analysis for Maternal Depressive Symptoms

**FIGURE 2:**
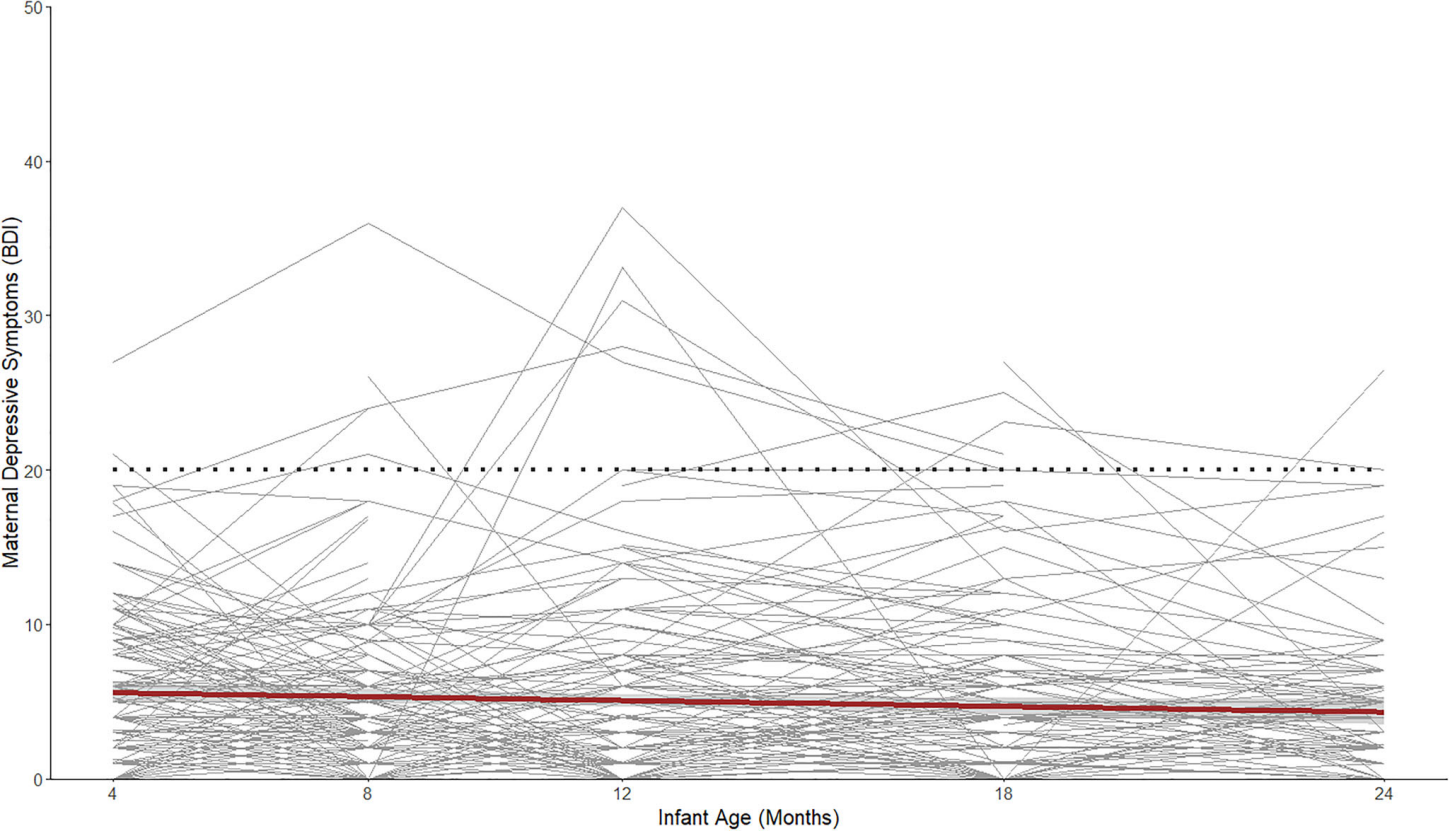
Individual and average trajectories of maternal depressive symptoms across infancy. Scores of 20 or higher (dotted line) indicate depression for nonclinical samples on the Beck's Depression Inventory (BDI).

A linear LGC model fit trajectories in maternal depressive symptoms well (*χ* ^2^(14) = 20.99, *p* = 0.10, CFI = 0.96, RMSEA = 0.04, SRMR = 0.09). In addition, the linear model did not significantly differ from a model including a quadratic term (*χ* ^2^(1) = 2.53, *p* = 0.11), indicating that the linear model was a more appropriate and parsimonious fit to the data. Figure 2 illustrates the change trajectory over time.

This linear growth model, constrained for measurement equivalence (e.g., equal measurement error across timepoints), indicated that, across the group, initial levels (estimate = 1.97, *p* < 0.01) and linear change over time (estimate = −0.08, *p* < 0.01) were significantly different from zero. Additionally, mothers had significant variability in their initial levels of depressive symptoms (estimate = 0.99, *p* < 0.01). However, there was no significant variation between individuals in change in maternal symptoms across infancy in the present sample (estimate = 0.008, *p* = 0.54) and no significant covariance between initial levels and linear change (estimate = 0.02, *p* = 0.52). Model results remained consistent when including maternal education as an auxiliary variable to help account for missingness. Based on the lack of individual differences in change in maternal depressive symptoms during infancy, the measure was reduced to a composite average score for the final model for parsimony.

#### 3.3.2 Unconditional Growth Curve Analysis for Infant Resting RSA

**FIGURE 3:**
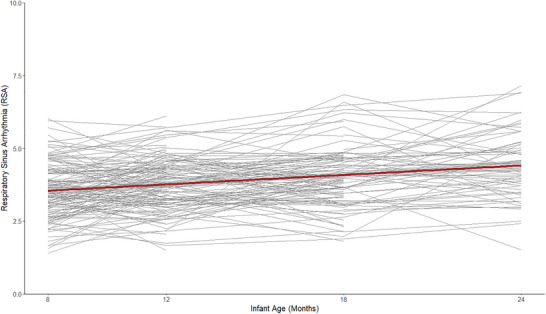
Individual and average infant RSA trajectories.

A linear LGC model fit trajectories of infant RSA well (*χ* ^2^(8) = 7.98, *p* = 0.44, CFI = 1.00, RMSEA = 0.00, SRMR = 0.06) and did not significantly differ from a model including a quadratic term (*χ* ^2^(4) = 0.02, *p* = 0.99). Figure 3 illustrates the linear change in RSA over infancy. This linear growth model, constrained for measurement equivalence, indicated significant initial levels of resting RSA at 8 months (estimate = 3.57, *p* < 0.01), significant average increases in resting RSA across infancy (estimate = 0.21, *p* < 0.01), significant variation in initial levels of RSA (estimate = 0.38, *p* < 0.01), as well as significant variation in linear change across infancy between subjects (estimate = 0.03, *p* = 0.02).

#### 3.3.3 SEM for Mediating Effect of Maternal Depression on Neighborhood to Infant RSA

**FIGURE 4:**
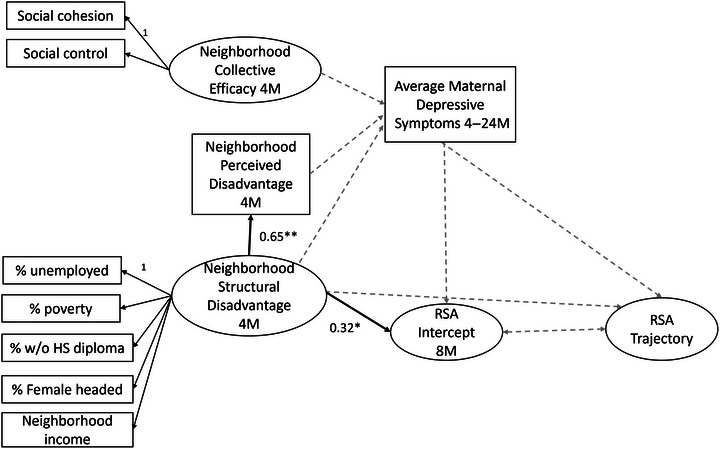
Mediation model results. **p < 0.01; *p < 0.05. Solid black lines indicate statistically significant regression paths, whereas gray dashed lines indicate nonsignificant paths (*p* > 0.05).

The full proposed model as indicated in Figure 1 accounting for maternal education and collapsing to a composite for maternal symptoms adequately fit the data (Robust *χ* ^2^(81) = 140.59, *p* < 0.01, CFI = 0.94, RMSEA = 0.05, SRMR = 0.09). However, the interaction term was not significantly related to any of the outcome measures and was therefore dropped as described in the missing data analysis results. The model without the interaction term provided an adequate fit to the data (Robust *χ* ^2^(70) = 105.04, *p* < 0.01, CFI = 0.96, RMSEA = 0.04, SRMR = 0.06), which was significantly better than the model including the interaction (*χ* ^2^(11) = 33.88, *p* < 0.01). Results from this model are presented in Figure 4 and Table 2.

**TABLE 2:** Estimates of model parameters and indirect effects.

		Std. estimate	Std. Err.	*p*	95% CI
		Factor loadings			
Structural disadvantage					
	Percent unemployed	0.61	0.07	0.000	[0.47, 0.75]
	Percent without HS diploma	0.67	0.04	0.000	[0.59, 0.76]
	Percent below poverty	0.86	0.03	0.000	[0.79, 0.92]
	Percent female headed	0.86	0.03	0.000	[0.79, 0.93]
	Neighborhood income	−0.71	0.03	0.000	[−0.78, −0.64]
Collective efficacy					
	Perceived cohesion	0.90	0.05	0.000	[0.80, 1.00]
	Perceived social control	0.74	0.05	0.000	[0.64, 0.85]
Outcome	Predictors	Regression slopes			
Perceived disadvantage					
	**Structural disadvantage**	**0.65**	**0.08**	**0.000**	**[0.48, 0.81]**
Mat. depression composite					
	Structural disadvantage	−0.12	0.16	0.456	[−0.73, 0.19]
	Perceived disadvantage	0.03	0.13	0.827	[−0.23, 0.29]
	Collective efficacy	−0.18	0.12	0.132	[−0.41, 0.05]
Initial infant RSA					
	**Structural disadvantage**	**0.32**	**0.13**	**0.018**	**[0.05, 0.58]**
	Mat. depression composite	0.03	0.11	0.773	[−0.18, 0.24]
Slope infant RSA					
	Structural disadvantage	0.25	0.24	0.287	[−0.21, 0.72]
	Mat. depression composite	−0.02	0.21	0.921	[−0.44, 0.39]
		Indirect effects			
Str. Dis. → Perc. Dis. → Mat. Dep. → Initial RSA		0.00	0.00	0.858	[−0.01, 0.01]
Str. Dis. → Perc. Dis. → Mat. Dep. → Slope RSA		0.00	0.00	0.927	[−0.01, 0.01]
		Covariances			
Initial infant RSA	Slope infant RSA	−0.17	0.25	0.497	[−0.65, 0.31]
Maternal education	Mat. depression composite	0.01	0.05	0.877	[−.09, 0.11]
	Initial infant RSA	0.01	0.10	0.960	[−0.20, 0.21]
	Slope infant RSA	−0.01	0.20	0.975	[−0.40, 0.40]
	Structural disadvantage	−0.66	0.05	0.000	[−0.76, −0.56]
	Perceived disadvantage	−0.05	0.08	0.513	[−0.20, 0.10]
	Collective efficacy	0.38	0.07	0.000	[0.25, 0.51]
		Intercepts			
Initial infant RSA		5.73	0.62	0.000	[4.52, 6.93]
Slope infant RSA		1.20	0.40	0.002	[0.43, 1.98]
		Variances			
Meas. error infant RSA		0.46	0.06	0.000	[0.33, 0.57]
Structural disadvantage		1	0	N/A	N/A
Collective efficacy		1	0	N/A	N/A
Perceived disadvantage		0.58	0.11	0.000	[0.37, 0.79]
Mat. depression composite		0.97	0.04	0.000	[0.90, 1.05]
Initial infant RSA		0.90	0.09	0.000	[0.73, 1.07]
Slope infant RSA		0.93	0.12	0.000	[0.70, 1.17]

*Note:* Bolded values highlight *p* < 0.05 in regression effects. Standard errors bootstrapped with 1000 draws.

This model indicated a significant positive association between neighborhood structural disadvantage and perceived neighborhood disadvantage (*β* = 0.65, *p* < 0.01, 95% CI [0.49, 0.80]). In addition, the model indicated a significant positive association between neighborhood structural disadvantage and initial levels of infant resting RSA (*β* = 0.32, *p* < 0.01, 95% CI [0.09, 0.55]). To test the mediating role of maternal depressive symptoms, indirect effects were estimated and presented in Table 2. Results indicated no significant indirect effects of structural disadvantage on initial levels or trajectories of infant resting RSA through maternal depressive symptoms.

#### 3.3.4 Exploratory Analyses

Given the lack of association between maternal symptoms and neighborhood disadvantage metrics, we sought to further explore if the association between initial resting RSA at 8 months and structural neighborhood disadvantage held beyond the complex covariance structure of the full model. Information on the specification of exploratory models is provided in Supporting Information S2. When regressing intial levels of infant RSA at 8 months on latent neighborhood structural disadvantage, controlling for maternal education, the model provided good fit to the data (Robust *χ* ^2^(34) = 52.94, *p* = 0.02, CFI = 0.97, RMSEA = 04, SRMR = 0.05), and the resulting association between neighborhood structural disadvantage and intial infant resting RSA at 8 months remained significantly different from zero. Infants in neighborhoods facing greater structural disadvantage had higher resting RSA at 8 months above and beyond our proxy for socioeconomic status (*β* = 0.28, *p* = 0.02, 95% CI [0.05, 0.50]). Of note, we ran an additional exploratory test of nonlinear relations between neighborhood structural disadvantage and infant resting RSA. This model provided a poor fit to the data (Robust *χ* ^2^(41) = 375.95, *p* < 0.01, CFI = 0.67, RMSEA = 0.16, SRMR = 0.17) and was therefore not pursued further (Kline 2016).

## 4 Discussion

This study investigated the mediating role of maternal depressive symptoms in the relation between neighborhood characteristics and infant PNS development. Our findings indicate that variability in infant resting RSA over the first 2 years of life provides potential room for environmental influence on trajectories. Unexpectedly, we did not find a significant change in maternal depression symptoms over time, limiting our ability to capture interactive influences across this early developmental window. Therefore, we could no longer assess our hypotheses on the bidirectional influence of infant physiology on changes in maternal symptoms. However, our model was still able to assess our core research questions of the impact of distal neighborhood factors on infant parasympathetic development. We were also still able to assess the potential mediating role of maternal depressive symptoms with the caveat that average levels of maternal depressive symptoms no longer established temporal precedence in predicting infant RSA outcomes, and thus mediating effects should be interpreted as relational rather than causal. Contrary to our hypotheses, our results indicated a positive direct effect of neighborhood structural disadvantage on initial levels of infant RSA at 8 months with no mediating effects of maternal depressive symptoms.

### 4.1 Growth Trajectories of Maternal Depressive Symptoms and Infant RSA

In line with prior literature and our hypotheses, maternal depressive symptoms showed significant between‐person variation in initial levels at 4 months (Wu et al. 2011). Additionally, mean levels of maternal depressive symptoms at 4 months were also significantly different from zero. This significant variation in intercept shows that mothers of 4‐month‐old infants are heterogeneous in their depressive symptoms, potentially leading to variable trajectories that could emerge from the first months of life. In addition, consistent with prior literature, mothers on average showed a decrease in depressive symptoms across the study duration (Wu et al. 2011). However, they did not show significant between‐person differences in this trajectory. This was surprising given prior work indicating that there is often variability in change trajectories of maternal depression in infancy, which in turn may be related to infant outcomes (Wu et al. 2011).

Lack of variability in trajectories, despite variation in initial levels at 4 months, could point toward rank‐order stability in maternal depressive symptoms across our sample. This could be due to generally low levels of depressive symptoms across the sample, meaning individuals did not have much room to show further decreases in depressive symptoms as their scores were near floor levels. While ICCs indicated substantial variability between timepoints, when a person's average is low, relatively small deviations may result in larger within‐person variation proportions even though these deviations may not be conceptually meaningful. This lack of variability may also be due to the sampling method for the present study. The current sample was not recruited for variability in postpartum or maternal depressive symptoms in infancy and, in general, indexed low nonclinical levels of depression symptoms, with only 3% of the sample reporting above the questionnaire‐indicated threshold for depression at any timepoint (see Figure 2).

Results indicated significant variability in resting RSA at 8 months, overall sample increases in RSA across infancy, as well as significant variability in these increases from 8 to 24 months. These results replicate prior research showing increases in vagal tone across the first 3 years of life and variability in these increases (Bar‐Haim et al. 2000; Dollar et al. 2020; Porges and Furman 2011; Wagner et al. 2021). While resting RSA has been found to be relatively stable from 6 months to 5 years in low‐income Latino children (Alkon et al. 2011), the present study found variation in linear slopes across time. This difference in results may be due to differences in sample characteristics, where the present sample contained a broader range of income and racial/ethnic diversity. Both the present study and Alkon et al. (2011) were constructed to assess different primary research questions, as we investigated differences across contexts, while they focused on patterns within a specific sample. Together, the differences in findings broaden our understanding of resting RSA developmental change within infancy, rather than from infancy to childhood, and include more data collection points during the infancy period. Infant resting RSA is thought to increase rapidly across infancy due to increases in myelination of the vagus nerve, as well as development and strengthening of the stress response system; therefore, more intensive sampling may have aided us in detecting variability during this time of rapid change (Bar‐Haim et al. 2000; Dollar et al. 2020).

Variation in the magnitude of these increases may be due to differential exposure to stressors that may calibrate the stress response system (Del Giudice et al. 2011) or, potentially, due to individual differences in genetic factors that contribute to the development of the vagus nerve (Boyce and Ellis 2005). Further, individuals’ initial levels of RSA at 8 months were not related to their rate of change in RSA across infancy. This association may support the hypothesis that changes over time may be due to differential experiences in infancy, as it was not only those infants with lower initial RSA who showed greater increases, which would have indicated a catch‐up or ceiling effect, had it occurred.

### 4.2 Neighborhood Collective Efficacy and Maternal Depression

One of the aims of the present study was to test how neighborhood perceived collective efficacy influenced maternal depressive symptoms across infancy, controlling for structural factors as well as infant factors. Prior work has found support for a negative association between neighborhood social support and maternal depressive symptoms (Kim and Ross 2009; Manuel et al. 2012; McCloskey and Pei 2019). In accordance with this work and pluralistic neighborhood theory (Aber and Nieto 2000), we hypothesized that collective efficacy would moderate the relation between neighborhood perceived disadvantage and maternal depressive symptoms. Results from the present study did not support this hypothesis and indicated no direct effect of collective efficacy, nor any interaction effects with neighborhood perceived disadvantage on average levels of maternal depressive symptoms across infancy.

Prior studies investigating associations between neighborhood characteristics and maternal depressive symptoms included larger samples from urban areas with larger ranges in demographic characteristics and maternal depressive symptoms than are present in the current sample (Manuel et al. 2012; McCloskey and Pei 2019). The current study, in contrast, sampled from three cities and has a higher mean income and maternal income, as well as lower racial/ethnic diversity. Additionally, as indicated in the correlations in Table 1, only neighborhood social cohesion was significantly correlated with maternal depressive symptoms at the concurrently assessed 4‐month timepoint. The other component of collective efficacy, informal social control, indexes the likelihood of neighbors to monitor youth behavior and neighborhood problems, such as skipping school and vandalism. Informal social control was not significantly correlated with maternal depressive symptoms in this sample. Prior work has indicated that informal social control and social cohesion may be contributing differentially to outcomes such as parenting practices and thus should be investigated separately (Zuberi 2016). Alternatively, social neighborhood cohesion may be a predictor of informal social control, and combining the constructs may not correctly represent social processes occurring within the neighborhood (Hipp and Wo 2015). In the present study, we proceeded with our a priori proposed analysis by including the combined collective efficacy construct in the final model, but future studies may consider teasing apart these neighborhood social factors when investigating their influence on maternal and child outcomes. In follow‐up post hoc analyses, moderated mediation structural equation models including only social cohesion or informal social control rather than the combined collective efficacy construct did not provide a good fit to the data in the present sample.

Prior work has theorized that informal social control may be related to lower levels of depression in adults by instilling a sense of neighborhood safety (Ross 2000; Wandersman and Nation 1998). In the present sample of mothers of infants, it is possible that social control, while it contributes to collective efficacy, may not impart the same sense of safety or decreased neighborhood stress to parents of young infants. Younger infants have no autonomy in navigating their neighborhood. This lack of autonomy allows mothers more control in infants’ exposure to their environments, which may contribute to a decreased effect of neighbors’ social control on maternal mental health. When social cohesion and social control were combined in the collective efficacy construct, we did not see an association with maternal depressive symptoms in the present sample.

### 4.3 Mediating Effects of Maternal Depression on Infant RSA

The main aim of the present study was to test the mediating role of maternal depression in the effects of neighborhood on infant PNS development. Results indicated a positive direct association between neighborhood structural disadvantage and infant resting RSA at 8 months, even after controlling for average maternal depressive symptoms as well as maternal education. This was contrary to our hypothesis that neighborhood structural characteristics would only have an indirect association with infant RSA through maternal depressive symptoms. Furthermore, the positive association between neighborhood structural disadvantage and 8‐month resting RSA was contrary to our expectations.

Individual differences in PNS development were theorized to be related to adaptive calibration of the stress response system to the environmental context (Del Giudice et al. 2011). Hypotheses generated by this model assert that basal levels of PNS engagement, commonly indexed by resting RSA, will decrease as environmental stress increases. Contrary to this hypothesis, results of the present study suggest that increased levels of structural disadvantage are related to higher resting levels of RSA, indicating a higher capacity to regulate in response to stress at 8 months of age. This contrasts with prior work that has found negative associations between resting RSA and neighborhood disadvantage in older children with similar demographic characteristics (Feurer et al. 2020).

Lastly, the study hypothesized that neighborhood disadvantage would indirectly affect trajectories of infant RSA through maternal depressive symptoms, with higher levels of neighborhood disadvantage related to less increases in infant RSA over time. Results from the current study indicated no indirect association between neighborhood disadvantage and initial levels of infant resting RSA through maternal depressive symptoms. Furthermore, results also indicated no indirect association between neighborhood disadvantage and linear changes in resting RSA across infancy through maternal depressive symptoms.

The positive association between neighborhood and infant RSA was contrary to our hypothesis of a negative association, which may be related to the age of the present sample. Other studies investigating relations between neighborhood characteristics and childhood regulation have focused on outcomes in toddlerhood or early childhood rather than early infancy (Kohen et al. 2008; Zhang et al. 2018). In addition, the present sample's age and demographic characteristics may be generally marked by low levels of environmental stress. While the ACM posits that increased environmental stress may relate to the development of lower basal parasympathetic engagement, prior work has found that lower levels of resting RSA may confer biological sensitivity to context in low‐risk samples (Suurland et al. 2018). Therefore, in contradiction to the hypothesis of the ACM, but in line with the theoretical framing, the PNS system may calibrate to lower resting RSA levels in contexts marked by the lowest levels of environmental risk. This adaptation, in turn, may potentiate a “sensitive profile” that is more responsive to changes in the environment.

In contexts marked by moderate levels of environmental stress, the stress response system develops to account for an environment that may include both occasional stressors and supportive parenting, resulting in a greater capacity to regulate. In low‐stress neighborhoods, the stress response system may not have these same strengthening opportunities, resulting in a lower overall capacity to regulate, as regulation is not needed in daily life. While the ACM indicates linear associations between environment and resting levels and reactivity of the PNS, it asserts a U‐shaped association with engagement and reactivity of the SNS. It remains untested whether this U‐shaped association may in fact also be present in associations between the PNS and the environment. In our current sample, an exploratory analysis of the potential U‐shaped association between neighborhood characteristics and infant RSA provided a poor fit to the data. Future studies containing samples with broader ranges of neighborhood characteristics may explore potential subgroups of neighborhood characteristics to further elucidate these potential U‐shaped associations with RSA exhibited in other empirical studies examining the effect of stress on developmental outcomes (Del Giudice et al. 2011; Oshri et al. 2022). Furthermore, while maternal depressive symptoms have been directly related to lower levels of infant RSA in other research (Curci et al. 2023; Propper and Holochwost 2013), no prior study has investigated the mediating role maternal depression may play in conveying the influence of neighborhood context on RSA outcomes. Given that infant RSA was still significantly positively related to neighborhood structural disadvantage both in the presence and absence of mediating effects in our exploratory analyses, the present study cannot rule out additional mediating factors that may be influencing the relation.

Parenting practices have been shown to be influenced by neighborhood characteristics and to relate to variation in infant RSA development in infancy and early childhood (M. Johnson et al. 2017; Kohen et al. 2008). Future work may seek to focus on how caregiver behavior or interactions with the neighborhood may mediate the relations between neighborhood and infant PNS development in this younger age range.

### 4.4 Limitations and Future Directions

It is worth noting that a large portion of the sample—27% at the 18‐month timepoint and 44% at the 24‐month timepoint—was collected after the onset of the COVID‐19 pandemic. Recent work has illustrated the impact of the pandemic on maternal depressive symptoms and, across samples, has found increased levels of maternal and postpartum depression after the onset of the pandemic (Hessami et al. 2020; Perzow et al. 2021; Racine et al. 2021). However, the current sample showed no significant variation in decreasing linear trajectories of maternal depression from 4 to 24 months of infant age, nor did it show a significant quadratic trend. This indicates that members of the sample showed no differences in their trajectories, and thus, we simplified to a composite score for maternal depressive symptoms across infancy. In a follow‐up *t*‐test, we found no significant differences in average maternal depressive symptoms in those who completed all timepoints before the onset of the pandemic (*M* = 1.86) compared to those who completed one or more timepoints after the onset of the pandemic (*M* = 1.78, *t*(282) = 0.49, *p* = 0.63).

The present analysis did not account for the possibility of participant residential mobility or general neighborhood residential mobility. In the case of participant mobility, this could lead to additional measurement error in neighborhood characteristics in the present sample, as neighborhood data were only calculated from addresses at the 4‐month timepoint. Regarding general neighborhood residential mobility, prior work has highlighted the role that residential mobility plays in the stability in one's neighbors, which may, in turn, influence neighborhood social support characteristics. While research has shown that residential mobility may affect adolescent outcomes (Witherspoon et al. 2009), future work should examine potential effects on support systems for mothers of infants and on infants themselves.

## 5 Conclusions

In summary, given the rapid development in infancy and the importance of resting RSA for behavioral outcomes later in life, the current study explored potential contextual factors that contribute to variability in the development of resting RSA across the first 2 years of life. Results from this study align with prior growth trajectories of maternal depression and infant RSA and contribute to possible neighborhood influences on infant regulatory development. Neighborhood context is often ignored when investigating infant development, despite having potential indirect effects through the family microsystem. The present study illustrates the importance of going beyond the proximal context to consider neighborhood effects in infant research and has found positive associations between census‐derived structural neighborhood disadvantage and infant resting RSA at 8 months. Given the associations between neighborhood structure and initial infant resting RSA, the present study also highlights the importance of continuing research investigating alternative mediating mechanisms between neighborhood contexts and early infant RSA development for potential intervention.

## Conflicts of Interest

The authors declare no conflicts of interest.

## Supporting information




**S.1**. Confirmatory Factor Analysis for Neighborhood Structural Disadvantage
**S.2**. Exploratory Direct Effects of Neighborhood Structural Disadvantage on Resting RSA.

## Data Availability

The data and code that support the findings of this study are openly available on Databrary at http://doi.org/10.17910/b7.1288.
