# Supplementary material for: The Influence of Neighborhood on Infant Parasympathetic Nervous System Development
Source: Dev Psychobiol. 2025 Sep 2;67(5):e70074. doi: 10.1002/dev.70074 (PMC12405823; doi:10.1002/dev.70074)
Supplement: Supplementary file 1 — S.1. Confirmatory Factor Analysis for Neighborhood Structural Disadvantage S.2. Exploratory Direct Effects of Neighborhood Structural Disadvantage on Resting RSA. [file DEV-67-e70074-s001.docx]

**Supporting Information**

**S.1. Confirmatory Factor Analysis for Neighborhood Structural Disadvantage**

A one-factor model fit the five neighborhood measures well (χ2(5) = 11.139 , *p* = 0.05, CFI = 0.989, RMSEA=.070, SRMR=.026). All variables significantly loaded onto the neighborhood structural disadvantage factor. Neighborhood income loaded negatively as expected while all other variables (percent residents unemployed, percent residents below the poverty line, percent of female headed households, and percent of residents without a high school diploma) loaded positively onto the latent factor. This latent factor structure was included in the final structural equation model.

**S.2. Exploratory Direct Effects of Neighborhood Structural Disadvantage on Resting RSA**

Exploratory analyses leveraged the same model specified in the unconditional linear latent growth curve model for infant resting RSA. The first exploratory model investigated the direct effect of neighborhood structural disadvantage on initial levels of infant resting RSA at 8-months and linear change in infant resting RSA from 8 to 24 months, controlling maternal education. The second exploratory model further included the quadratic effect of the latent neighborhood structural disadvantage as a predictor of initial levels and change in infant RSA. Due to software limitations in the ability to estimate the quadratic effect directly within the model, the quadratic effect of latent neighborhood disadvantage was approximated by exporting the individual latent factors from the confirmatory factor analysis (see S1) and squaring these individual scores.
